# Management of Spontaneous Subarachnoid Hemorrhage Patients with Negative Initial Digital Subtraction Angiogram Findings: Conservative or Aggressive?

**DOI:** 10.1155/2017/2486859

**Published:** 2017-05-02

**Authors:** Liang Xu, Yuanjian Fang, Xudan Shi, Xianyi Chen, Jun Yu, Zeyu Sun, Jianmin Zhang, Jing Xu

**Affiliations:** ^1^Department of Neurosurgery, The Second Affiliated Hospital, School of Medicine, Zhejiang University, Hangzhou, Zhejiang, China; ^2^Brain Research Institute, Zhejiang University, Hangzhou, Zhejiang, China; ^3^Collaborative Innovation Center for Brain Science, Zhejiang University, Hangzhou, Zhejiang, China; ^4^Department of Anesthesiology, The Second Affiliated Hospital, School of Medicine, Zhejiang University, Zhejiang, China

## Abstract

*Background.* The ideal management of SAH patients with negative initial DSA findings remains unresolved.* Objective.* (i) To present risk factors, clinical courses, and outcomes in different types of SAH patients with negative DSA findings; (ii) to explore the differences of basal vein between aSAH patients and NASAH patients; and (iii) to evaluate the value of repeated DSA for these patients.* Methods.* All SAH patients with negative initial DSA findings between 2013 and 2015 in our hospital were enrolled and were further categorized as perimesencephalic SAH (PMN-SAH) or nonperimesencephalic SAH (nPMN-SAH). Risk factors, clinical courses, outcomes, and the basal vein drainage patterns were compared.* Results.* A total of 137 patients were enrolled in the present study. The PMN-SAH group had better GOS and mRS values at 1-year follow-up. Moreover, the nPMN-SAH group had a higher rate of complications. The basal vein drainage pattern showed significant difference when comparing each of the NASAH subtypes with aSAH groups. There was a significant higher rate of a responsible aneurysm in nPMN-SAH group upon repeated DSA.* Conclusions.* SAH patients with negative initial DSA findings had benign clinical courses and outcomes. Repeated DSA studies are strongly advised for patients with the nPMN-SAH pattern.

## 1. Introduction

Spontaneous subarachnoid hemorrhage (SAH), characterized by bleeding into the subarachnoid space in the absence of trauma, is most often caused by the rupture of an intracranial aneurysm [1, 2]. However, according to the previous studies [3, 4], even though there is now widespread use of digital subtraction angiogram (DSA) to aid in the diagnosis of spontaneous SAH, nearly fifteen percent of cases remain idiopathic [4]. This category of SAH has been termed nonaneurysmal SAH (NASAH), which typically follows a benign clinical course and has a generally favorable prognosis when compared with aneurysmal subarachnoid hemorrhage (aSAH) [5, 6].

According to the distribution pattern of the subarachnoid blood, these NASAH patients are usually divided into two subcategories, perimesencephalic (PMN-SAH) and nonperimesencephalic hemorrhage (nPMN-SAH) [7]. Although it is generally recognized that NASAH has a more preferable outcome than aSAH [5], recent studies suggest that the management of the nPMN-SAH subgroup should be more rigorous in light of its more severe clinical courses and outcomes [8, 9]. As nPMN-SAH is a diagnosis of exclusion, some controversial issues remain regarding the management of these patients that have negative initial DSA findings [10, 11]. Despite numerous studies utilizing multiple imaging modalities, the bleeding source of NASAH has not been elucidated. Though most authors support the hypothesis that the culprit of the bleeding is of venous origin, the precise mechanism of the bleeding source remains unknown [12]. Furthermore, given the low detection rate of a responsible intracranial lesion in NASAH patients, the need for serial DSAs in these cases remains an open question.

Here we retrospectively analyzed the data of all SAH patients with initial negative DSA findings in our center between 2013 and 2015. The risk factors, clinical courses, and outcomes of these patients were evaluated in our study. In addition, the venous drainage patterns in both subgroups were compared. Finally, the necessity of repeated DSA examinations was also evaluated based on our study and the review of related literature.

## 2. Methods

### 2.1. Patients and Procedure

**Figure 1:**
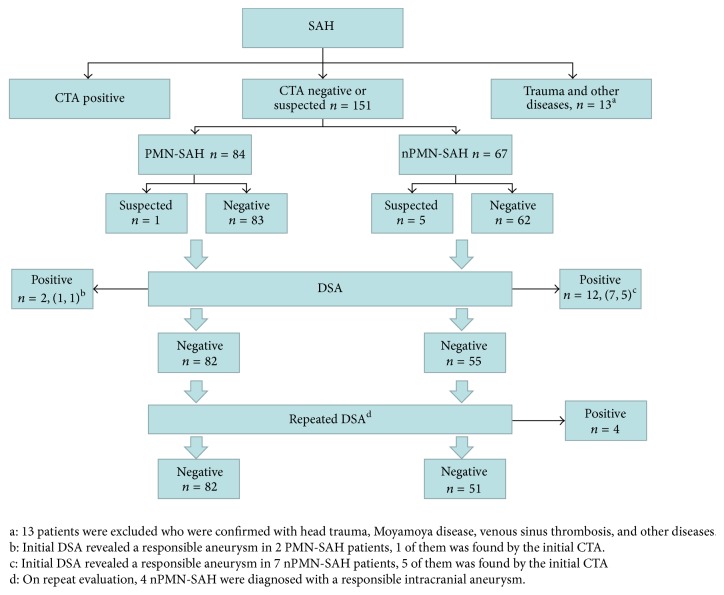


The data of patients who presented with SAH between 2013 and 2015 in our center (Second Affiliated Hospital, School of Medicine, Zhejiang University) were retrospectively analyzed. According to standard management of SAH patients [13, 14], all patients were screened by CTA upon admission, followed by emergent DSA examination. There were 164 SAH patients who had an initial CTA that failed to definitively demonstrate a culprit lesion. Of these, 13 were excluded due to a history of traumatic brain injury or another definitive cause of SAH, leaving 151 patients with negative CTA findings. Among them, 14 patients had positive initial DSA results (2 with a perimesencephalic pattern and 12 with a nonperimesencephalic pattern). This left 137 SAH patients with negative initial DSA findings (hereafter referred to as NASAH patients) who were enrolled in the present study. Based on the distribution of the subarachnoid blood, NASAH patients were divided into PMN-SAH (*n* = 82 patients) and nPMN-SAH groups (*n* = 55) [15]. To further exclude intracranial aneurysm as the source of hemorrhage [16], all of them underwent a repeated DSA examination either 10–14 days after admission or one month after discharge. In total, 57 patients agreed to undergo a repeated DSA during hospitalization or follow-up. Four patients were found to have a culprit intracranial aneurysm on the second angiogram, all of whom were patients with the nonperimesencephalic pattern (Figure 1).

The demographic data includes patient sex, age, smoking history, alcohol use, hypertension, diabetes, and history of anticoagulant use. The Glasgow Coma Score (GCS), Hunt-Hess (HH) grade, and the modified Fisher Scale (mFS) were used for evaluation upon admission [5, 33]. The length of hospital stay (LOS) and in-hospital complications, namely, hydrocephalus, cerebral vasospasm, and rebleeding, were compared between the two groups. All patients were followed up by telephone interview or outpatient clinic at three months and one year after discharge. The outcomes were evaluated using the modified Rankin Scale (mRS) and Glasgow Outcome Scale (GOS).

In order to find a potential venous source of bleeding, we compared the basal vein of Rosenthal (BVR) anatomy among the different groups. Excluding 4 patients who were later found to have a definite bleeding source, the venous configurations of 133 NASAH patients (82 PMN-SAH and 51 nPMN-SAH) were evaluated and compared to a total of 133 consecutive aSAH patients during the same period. The classification of the BVR was performed as described by Ramazan Buyukkaya et al. [12]. Briefly, the drainage pattern of unilateral BVR was divided into three types:

1. Type A (normal continuous): a continuous BVR drains mainly into the vein of Galen.
2. Type B (normal discontinuous): in a discontinuous BVR, the anterior part drains into the sphenoparietal sinus or cavernous sinus through an uncal vein and posterior part drains to the vein of Galen.
3. Type C (primitive variant): this variant drains mainly to the dural sinuses, not via the vein of Galen (i.e., the perimesencephalic veins drain into the superior petrosal sinus or the BVR drains directly into the transverse or straight sinus).

### 2.2. Statistical Analysis

Categorical variables of different groups, such as the parameter of demographic data, GCS, HH grade, mFS, mRS, and GOS, were compared using the Chi-squared test or Fisher's exact test. Continuous variables, such as the number of patients in each group, were compared using Student's *t*-test. *p* < 0.05 was considered as significant statistical difference.

## 3. **Results**

### 3.1. Demographics

The demographics of all NASAH patients with negative initial DSA findings are summarized in Table 1. There were no significant differences in sex, age, smoking history, alcohol abusing history, diabetes, hypertension, or anticoagulation medication history between PMN-SAH and nPMN-SAH groups (*p* > 0.05 for each parameter).

### 3.2. Clinical Course and Risk Factors for Hydrocephalus

The patients' GCS, HH scale, and mFS were evaluated upon arrival at our emergency room. Despite the interval from ictus to evaluation, the nPMN-SAH group presented with higher GCS and mFS on their initial CT scan (*p* < 0.05). However, there was no significant difference in the HH grade between the two groups (*p* = 0.157) (Table 2).

Complications that developed during hospitalization, hydrocephalus, cerebral vasospasm, and rebleeding were compared. The nPMN-SAH group was associated with higher incidence of hydrocephalus and symptomatic cerebral vasospasm (*p* = 0.001 and 0.017, resp.). Only one patient experienced rebleeding but no definite source of bleeding was confirmed even though a repeat DSA was conducted. There was no significant difference in the rebleeding rate during hospitalization between the two groups (*p* = 0.401) (Table 2).

There were 14 patients (10.2%) who developed hydrocephalus. The risk factors for hydrocephalus in these patients were compared. There were significant differences in the GCS and mFS between the hydrocephalus group and nonhydrocephalus group (*p* < 0.001). Furthermore, patients who developed hydrocephalus were associated with significantly higher proportion of a diffuse bleeding pattern on imaging studies (e.g., nPMN-SAH and intraventricular hemorrhage) (*p* = 0.002 and *p* < 0.001, resp., Table 3).

### 3.3. Outcome

A total of 21 patients (12 PMN-SAH and 9 nPMN-SAH) were lost to follow-up after discharge. The mRS and GOS were used to evaluate the outcomes for all patients. We found that patients with PMN-SAH had a better clinical outcome at 3 months after ictus with respect to both mRS (*p* < 0.001) and GOS (*p* = 0.003) (Table 4). Though there was no significant difference of GOS between the two groups at 1-year follow-up (*p* = 0.09), the nPMN-SAH group had a trend towards a higher incidence of mild disability (mRS = 2 or 3, *p* = 0.006, Table 4).

### 3.4. BVR Pattern

The venous phase images on DSA for 3 NASAH patients were unavailable due to technical reasons. The distribution of BVR subtypes of three groups are listed in Table 5. Compared with aSAH patients, there was a significant difference of BVR distribution in both of the PMN-SAH group (*p* = 0.003) and nPMN-SAH group (*p* = 0.021). However, there was no statistically significant difference of unilateral BVR development between PMN-SAH and nPMN-SAH groups (*p* = 0.950). To further analyze the correlation of hemorrhage type and BVR development, the bilateral BVRs were classified as four subgroups, AA, AB/BB, AC/BC, and CC, respectively. A significant statistical difference was only found between PMN-SAH and aSAH groups (*p* = 0.028, Table 6).

### 3.5. Repeated DSA Findings

To evaluate the utility of repeated DSA examination in patients with initially negative DSA findings, we compared the detection rate of a responsible intracranial lesion by the repeated DSA in both of the subgroups and also included data from related literature for analysis. A total of 60 patients underwent a repeated DSA examination in our series. On repeat evaluation, 4 of 31 patients with nPMN-SAH were diagnosed with a responsible intracranial aneurysm, resulting in a detection rate of 12.9% on repeat DSA examination; however none of the PMN-SAH patients had positive findings on their repeated DSA (Table 7). According to the data from recent studies, the detection rate of a responsible intracranial aneurysm by repeated DSA was 12.5% in the nPMN-SAH subgroup and 1.2% in the PMN-SAH subgroup (Table 7).

## 4. **Discussion**

SAH patients with negative initial DSA findings, usually defined as NASAH, can be a management challenge as the optimal management scheme for these patients remains controversial. The present study analyzes the data of SAH patients with negative initial DSA findings from our center. We divided those patients into the two subgroups of PMN-SAH and nPMN-SAH. The development of BVR type in each group was compared with an aSAH control group to help elucidate the source of bleeding. The necessity of repeated DSA examinations was evaluated in each group by calculating the positive finding rate from second angiogram.

It is wildly accepted that NASAH patients have a more favorable clinical course and a lower incidence of complications when compared to those with aSAH [34]. The subgroup of PMN-SAH was first described by van Gijn et al. [35] as a benign entity, characterized by the distribution of the subarachnoid hemorrhage mainly or only in the perimesencephalic cisterns. However, the nPMN-SAH group has a more diffuse distribution of the subarachnoid hemorrhage that is more similar to aSAH [15]. A diagnosis of nPMN-SAH is associated with higher rate of complications, such as hydrocephalus, cerebral vasospasm, and cerebral infarction [5]. Hydrocephalus is one of the most common complications of NASAH, normally identified by symptoms of elevated intracranial pressure (progressive headache, altered sensorium) or findings on head CT [36]. The incidence of hydrocephalus from the present cohort was 14.3%, which is in agreement with previous reports [34]. We also found that, in the nonperimesencephalic pattern, the presence of intraventricular hemorrhage or poorer clinical grade on presentation (GCS < 13 and modified Fisher Scale > 2) were associated with the development of hydrocephalus.

Interestingly, despite the repeated imaging studies that have been performed on NASAH patients, the etiology of bleeding remains obscure. The potential pathogenesis may include a venous system variant [20], capillary abnormality, intracranial basilar dissection, ruptured perforating artery [37], cavernous malformation [38], or capillary telangiectasia [15]. Although both arterial and venous origins for the SAH have been proposed, most of the studies favor a venous source of PMN-SAH [39]. It was first hypothesized by Watanabe et al. [17] that a large portion of the BVRs in PMN-SAH patients vary from a normal configuration with drainage into dural sinuses instead of the vein of Galen. Similarly, van der Schaaf et al. [19] reported that primitive venous drainage was more common in patients with PMN-SAH and that the venous drainage variation was ipsilateral to the side of bleeding. Most of the subsequent studies supported the theory that the drainage into the deep venous drain system (the vein of Rosenthal variant) was related to the PMN-SAH [12, 18–20, 22–24]. However, a study from Daenekindt et al. [21] suggested otherwise (Table 6), but this may be explained by its relatively small patient series, short follow-up period, or differences in diagnostic criteria. Similar to prior publications, our present study demonstrated a significant difference in BVR anatomy between the PMN-SAH and aSAH groups, regardless of unilateral or bilateral BVR type (*p* = 0.003, 0.028) and that the primitive venous configuration was ipsilateral to the source of the bleeding. However, it remains unclear exactly how the venous drainage variation contributes to PMN-SAH.

However, unlike with the PMN-SAH group, the relationship between venous drainage and nPMN-SAH was rarely described previously. It is generally hypothesized that the nPMN pattern may have resulted from an arterial source to account for its relatively “malignant” clinical presentation and extensive blood distribution into the cisterns and parenchyma. Consequently, this pattern of SAH is also referred to as aneurysm-like SAH. Interestingly, our study revealed that there was also a significant difference in the unilateral BVR type between the nPMN-SAH and aSAH groups (*p* = 0.021). However, for bilateral venous drainage, there was no significant difference between the two groups (*p* = 0.066). The inconsistent findings between the unilateral and bilateral BVR types could be explained by a contribution of true aSAH that were misclassified as nPMN-SAH because of an initial negative DSA. Although a higher portion of nPMN-SAH with negative initial DSA findings were eventually confirmed to have resulted from ruptured intracranial aneurysms, compared to PMN-SAH, most sources of nPMN-SAH remain idiopathic. Therefore, we conclude that if a ruptured intracranial aneurysm can be absolutely excluded, the bleeding source of a true nPMN-SAH may be similar to that found in PMN-SAH.

Another main concern in the management of NASAH is to evaluate the necessity of repeated DSA examinations for each individual. While DSA is currently the standard method to diagnose an intracranial aneurysm for patients suffering from SAH, there are risks to the procedure. Some studies have shown that catheter angiography has up to a 2.6% risk of permanent neurologic complications in NASAH [40]. Recently, numerous reports have proposed the use of noninvasive techniques to diagnose an intracranial aneurysm, such as CTA and magnetic resonance angiography (MRA) [41, 42]. The accuracy of the DSA result can be affected by numerous factors: the resolution of the DSA device, the quality of the acquired scan, the 3D reconstruction capabilities, the interval between the onset of the symptoms and the examination, and the experience of the technician. Furthermore, a small or dissecting aneurysm, hemorrhage or vasospasm concealing the aneurysm, or technical deficiencies can lead to a false-negative result upon the initial examination [43]. Even though improvements in imaging technology have decreased the incidence of misdiagnosis, there are still nearly 15% of SAH patients who have negative findings upon their initial DSA examination [43, 44]. Since missing an aneurysmal source on DSA would expose patients to the extensive morbidity and mortality of rebleed, most practitioners carry out repeat DSA exams on SAH patients with a negative initial DSA. Consequently, it would be of value to select the specific individuals who would benefit from repeated DSA exams.

According to our present study, a total of 60 patients underwent a repeat DSA examination. Four of 31 patients with nPMN-SAH were ultimately diagnosed with a ruptured intracranial aneurysm, resulting in a detection rate of 12.9% for a repeat DSA. However none of the PMN-SAH patients in our study had positive findings on their repeat DSA. To further address this finding, a thorough review of data from past studies was evaluated. There are 13 studies in the past 15 years that discuss a positive finding on repeat DSA exams in SAH patients with negative initial DSA findings. For the pooled data of PMN-SAH patients, a repeat DSA detected a culprit aneurysm in only 4 of 151 patients [2, 3, 8, 27, 28, 30, 32] (Table 7) [25, 26, 29, 31]. Combined with our data, the overall misdiagnosis rate is only 1.1%, which is likely lower than the risk of the DSA procedure itself. We therefore propose that it may be acceptable to follow up PMN-SAH patients by noninvasive image studies rather than DSA. Some authors go further, even raising the possibility that it is reasonable to manage PMN-SAH patients completely with noninvasive cerebral vascular imaging. The preference at our institution is that unless the initial CT scan with high quality vascular imaging is completed within several hours after the onset of symptoms, all PMN-SAH patients need at least one DSA to exclude a ruptured intracranial aneurysm. However, for centers without available neurointerventionalists, the DSA can be delayed because PMN-SAH is usually associated with a benign clinical course and good prognosis. Unlike the extremely low rate of aneurysm detection in PMN-SAH patients, our data revealed that 12.6% of nPMN-SAH patients were ultimately found to have a responsible aneurysm on the second DSA. In previous studies, the misdiagnosis rate of the initial DSA in nPMN-SAH patients varied greatly (misdiagnose rate from 4.7% to 45.9%), which is likely due to several factors, as previously mentioned. After pooling all the published studies, the overall misdiagnose rate was 12.5% (66 out of 527, Table 7), which is similar to the findings at our institution. Therefore, we strongly recommend a repeat DSA examination for patients with nPMN-SAH who had negative initial findings. Additionally, it is advisable to use 3D image acquisition for both internal carotid arteries and the vertebral arteries in the initial and repeat DSA to optimize the chances of finding small lesions. Since the aneurysms discovered on repeat imaging tend to be miniature in size and are found at the bifurcation of small perforating arteries, we also suggest consultation with an experienced neuroradiological or/and neurointervention physician to establish the diagnosis.

## 5. Conclusion

Managing SAH patients with negative initial DSA findings can be challenging. Based on the results of our present study and a review of the pertinent literature, the PMN-SAH subgroup usually has a benign clinical course and a repeat DSA very seldom reveals a ruptured intracranial aneurysm. More importantly, nPMN-SAH patients are associated with higher complication rate and incidence of a ruptured aneurysm. Therefore, we strongly recommend a repeat DSA in patients with nPMN-SAH pattern on initial imaging.

## Figures and Tables

**Table 1:** Demographics of patients with nonaneurysmal subarachnoid hemorrhage.

	NASAH	PMN-SAH	nPMN-SAH	*p* value
Sum (%)	137 (100%)	82 (59.9%)	55 (40.1%)	NS
Male-female ratio	76/61 (1.2/1)	42/40 (1.05/1)	33/22 (1.5/1)	0.357
Age (range)	56.0 ± 10.4 (30–80)	55.4 ± 9.6 (34–76)	56.7 ± 13.7 (30–80)	0.156
Smoker (%)	18 (13.1%)	11 (13.4%)	7 (12.7%)	0.907
Alcohol abusing (%)	21 (15.3%)	13 (15.9%)	8 (14.5%)	0.835
Diabetes (%)	7 (5.1%)	3 (3.7%)	4 (7.3%)	0.438
Hypertension (%)	30 (21.9%)	15 (18.3%)	15 (27.3%)	0.213
Anticoagulant Using (%)	4 (2.9%)	2 (2.4%)	2 (3.6%)	1

NASAH: nonaneurysmal subarachnoid hemorrhage, PMN-SAH: perimesencephalic nonaneurysmal subarachnoid hemorrhage, nPMN-SAH: nonperimesencephalic nonaneurysmal subarachnoid hemorrhage; NS means can not be analyzed.

**Table 2:** The clinical characteristics of nonaneurysmal subarachnoid hemorrhage.

	All NASAH (*n* = 137)	PMN-SAH (*n* = 82)	nPMN-SAH (*n* = 55)	*p* value
LOS, mean (range)	8.5 ± 8.5 (2–75)	7.7 ± 4.0 (2–16)	12.9 ± 11.9 (2–75)	0.001
GCS				
Mild (13–15)	132 (97.0%)	82 (100%)	50 (90.9%)	0.009
Middle (9–12)	1 (0.7%)	0	1 (1.8%)	
Severe (3–8)	4 (2.3%)	0	4 (7.3%)	
H-H grade				
Good (I-II)	132 (96.4%)	81 (98.8%)	51 (92.7%)	0.157
Poor (III-IV)	5 (3.6%)	1 (1.2%)	4 (7.3%)	
mFS				
0-1	78 (56.9%)	70 (85.4%)	8 (14.5%)	<0.001
2–4	59 (43.1%)	12 (14.6%)	47 (85.5%)	
Complication				
Hydrocephalus	14 (10.2%)	3 (3.7%)	11 (20.0%)	0.001
Cerebral vasospasm	7 (5.1%)	1 (1.2%)	6 (10.9%)	0.017
Rebleeding	1 (0.7%)	0	1 (1.8%)	0.401
Pulmonary infections	3 (2.2%)	1 (1.2%)	2 (3.6%)	0.564

NASAH: nonaneurysmal subarachnoid hemorrhage, PMN-SAH: perimesencephalic nonaneurysmal subarachnoid hemorrhage, nPMN-SAH: nonperimesencephalic nonaneurysmal subarachnoid hemorrhage, GCS: Glasgow coma scale, H-H grade: Hunt-Hess grade, mFS: modified Fisher Scale; LOS: length of hospital stay.

**Table 3:** Clinical characteristics of hydrocephalus patients in NASAH group.

	No hydrocephalus (*n* = 123)	Hydrocephalus (*n* = 14)	*p*
Demography			
Age	56.4 ± 10.0	52.9 ± 13.3	0.147
Gender (female)	57 (46.3%)	4 (28.6)	0.205
Smoker	16 (13.0%)	2 (14.3%)	0.893
Alcohol abuse	18 (14.6%)	3 (21.4%)	0.504
Hypertension	26 (21.1%)	4 (28.6%)	0.524
Diabetes	5 (4.1%)	2 (14.3%)	0.100
Clinical grade			
GCS < 13	0 (0%)	4 (28.6%)	<0.001
Modified Fisher Scale > 1	56 (45.5%)	11 (78.6%)	<0.001
Bleed Pattern			
PMN-SAH	79 (63.4%)	3 (21.4%)	0.002
nPMN-SAH	44 (35.8%)	11 (78.6%)	
Anterior circulation^a^	96 (78.0%)	12 (85.7%)	0.506
Posterior circulation^b^	27 (21.9%)	2 (14.3%)	
Intraventricular hemorrhage	8 (6.5%)	10 (71.4%)	<0.001
Cortical hemorrhage	22 (17.9%)	3 (21.4%)	0.745

EVD: external ventricular drain, GCS: Glasgow coma scale.

^a^Anterior circulation was characterized by blood mainly locating at anterior of the brain.

^b^Posterior circulation was characterized by blood mainly locating at posterior of the brain.

**Table 4:** The prognosis of patient after charged from hospital.

	All NASAH (*n* = 116)^*∗*^	PMN-SAH (*n* = 70)	nPMN-SAH (*n* = 46)	*p* value
mRS at 3 months				
0-1	96 (82.8%)	66 (94.3%)	30 (65.2%)	<0.001
2-3	17 (14.7%)	4 (5.7%)	13 (28.2%)	
4–6	3 (2.5%)	0	3 (6.6%)	
GOS at 3 months				
5	110 (94.8%)	70 (100%)	40 (87.0%)	0.003
4	3 (3.1%)	0	3 (6.5%)	
1–3	3 (3.1%)	0	3 (6.5%)	
mRS at 1 year				
0-1	78 (86.7%)	46 (95.8%)	32 (76.2%)	0.006
2-3	10 (11.1%)	2 (4.2%)	8 (19.0%)	
4–6	2 (2.2%)	0	2 (4.8%)	
GOS at 1 year				
5	87 (96.7%)	48 (100%)	39 (92.8%)	0.09
4	1 (1.1%)	0	1 (2.4%)	
1–3	2 (2.2%)	0	2 (4.8%)	

NASAH: nonaneurysmal subarachnoid hemorrhage; PMN-SAH: perimesencephalic nonaneurysmal subarachnoid hemorrhage; nPMN-SAH: nonperimesencephalic nonaneurysmal subarachnoid hemorrhage; GOS: Glasgow outcome scale, mRS: modified Rankin Scale.

^*∗*^21 patients (12 perimesencephalic and 9 nonperimesencephalic patients) were lost to follow-up after discharge.

**Table 5:** Type of BVR in nonaneurysmal subarachnoid hemorrhage and aneurysmal subarachnoid hemorrhage patients.

BVR type	PMN-SAH	nPMN-SAH	aSAH	*p*1	*p*2	*p*3
Unilateral	*n* = 160	*n* = 100	*n* = 266	0.003	0.021	0.950

A	61 (38.1%)	39 (39.0%)	147 (55.3%)			
B	48 (30.0%)	31 (31.0%)	61 (22.9%)			
C	51 (31.9%)	30 (30.0%)	58 (22.8%)			

Bilateral	*n* = 80	*n* = 50	*n* = 133	0.028	0.066	0.882

AA	15 (18.8%)	9 (18.0%)	50 (37.6%)			
AB, BB	26 (32.5%)	16 (32.0%)	38 (28.6%)			
AC, BC	26 (32.5%)	19 (38.0%)	32 (24.1%)			
CC	13 (16.2%)	6 (12.0%)	13 (9.8%)			

BVR: basal vein of Rosenthal; PMN-SAH: perimesencephalic nonaneurysmal subarachnoid hemorrhage; nPMN-SAH: nonperimesencephalic nonaneurysmal subarachnoid hemorrhage.

*p*1: statistical analysis was proceeded between PMN-SAH and aSAH group.

*p*2: statistical analysis was proceeded nPMN-SAH and aSAH group.

*p*3: statistical analysis was proceeded PMN-SAH and nPMN-SAH group.

**Table 6:** Type of BVR in nonaneurysmal subarachnoid hemorrhage and aneurysmal subarachnoid hemorrhage patients.

Author/year	Unilateral BVR type						Bilateral BVR type							
	PMN-SAH (%)			aSAH (%)			PMN-SAH (%)				aSAH (%)			
	A	B	C	A	B	C	AA	AB BB	AC BC	CC	AA	AB BB	AC BC	CC
Watanabe et al. [17]/2002	3 (25)	2 (17)	7 (58)	79 (41)	70 (37)	42 (22)	0	NS	NS	NS	22 (22)	NS	NS	NS
Alén et al. [18]/2008^*∗*^	36 (24)	66 (44)	47 (32)	116 (59)	51 (26)	31 (15)	12 (13)	41 (45)	38 (42)		54 (48)	34 (30)	24 (21)	
van der Schaaf et al. [19]/2008	21 (19)	48 (43)	21 (37)	49 (58)	26 (31)	9 (11)	4 (7)	NS	NS	29 (53)	15 (36)	NS	NS	8 (19)
Yamakawa et al. [20]/2008	10 (29)	7 (20)	18 (51)	111 (57)	61 (31)	23 (12)	2 (11)	NS	NS	NS	32 (29)	NS	NS	NS
Daenekindt et al. [21]/2008	49 (42)	34 (30)	32 (28)	50 (46)	31 (29)	27 (25)	13 (22)	18 (31)	28 (47)		10 (17)	28 (47)	21 (36)	
Song et al. [22]/2010	NS	NS	NS	NS	NS	NS	10 (31)	8 (25)	8 (25)	6 (19)	34 (60)	12 (21)	9 (16)	2 (4)
Kawamura et al. [23]/2011	6 (32)	8 (42)	5 (26)	33 (49)	27 (35)	7 (10.4)	0	5 (50)	3 (30)	1 (10)	14 (42)	12 (36)	7 (21)	
Sabatino et al. [24]/2014^*∗*^	36 (46)	31 (39)	12 (15)	50 (66)	8 (11)	18 (24)	NS	NS	NS	NS	NS	NS	NS	NS
Buyukkaya et al. [12]/2014	NS	NS	NS	NS	NS	NS	14 (35)	3 (9)	12 (34)	6 (17%)	26 (74)	5 (14)	3 (9)	1 (3)

PMN-SAH: perimesencephalic nonaneurysmal subarachnoid hemorrhage; aSAH: aneurysmal subarachnoid hemorrhage; NS means no data was recorded. ^*∗*^The idiopathic subarachnoid hemorrhage has not been described in detail; no group was divided.

**Table 7:** Diagnostic yield of a repeated DSA investigation.

Author/year	Study type	PMN-SAH	Positive	Misdiagnose rate	nPMN-SAH	Positive	Misdiagnose rate
Topcuoglu et al. [10]/2003	Retrospective	31	0	0	36	3	8.3%
Jung et al. [25]/2006	Retrospective	65	1	1.5%	37	17	45.9%
Huttner et al. [11]/2006	Prospective	38	0	0	NS	NS	NS
Little et al. [26]/2007	Retrospective	23	1	4.3%	59	5	8.5%
Gupta et al. [3]/2009	Retrospective	18	0	0	43	2	4.7%
Carvi y Nievas and Archavlis [27]/2009	Retrospective	8	0	0	3	1	33.3%
Agid et al. [9]/2010	Retrospective	28	0	0	28	4	14.3%
Fontanella et al. [28]/2011	Retrospective	23	0	0	72	9	12.5%
Maslehaty et al. [29]/2011	Retrospective	34	1	2.9%	120	13	10.8%
Kelliny et al. [30]/2011	Retrospective	35	0	0	37	6	16.2%
Delgado Almandoz et al. [31]/2012	Prospective	29	1	3.4%	39	2	5.1%
Lin et al. [32]/2012	Retrospective	27	0	0	41	2	4.9%
DW et al. [8]/2012	Retrospective	6	0	0	12	2	16.7%
Present study	Retrospective	31	0	0	29	4	13.8%
Total		365	4	1.1%	556	70	12.6%

PMN-SAH: perimesencephalic nonaneurysmal subarachnoid hemorrhage; nPMN-SAH: nonperimesencephalic nonaneurysmal subarachnoid hemorrhage; NS means no data was recorded.
